# Milk—A Nutrient System of Mammalian Evolution Promoting mTORC1-Dependent Translation

**DOI:** 10.3390/ijms160817048

**Published:** 2015-07-27

**Authors:** Bodo C. Melnik

**Affiliations:** Department of Dermatology, Environmental Medicine and Health Theory, University of Osnabrück, Osnabrück D-49090, Germany; E-Mail: melnik@t-online.de; Tel.: +49-5241-988-060; Fax: +49-5241-25-801

**Keywords:** aging, amino acids, ER stress, exosomes, diseases of civilization, microRNAs, milk signaling, mTORC1, quasi-program, translation

## Abstract

Based on own translational research of the biochemical and hormonal effects of cow’s milk consumption in humans, this review presents milk as a signaling system of mammalian evolution that activates the nutrient-sensitive kinase mechanistic target of rapamycin complex 1 (mTORC1), the pivotal regulator of translation. Milk, a mammary gland-derived secretory product, is required for species-specific gene-nutrient interactions that promote appropriate growth and development of the newborn mammal. This signaling system is highly conserved and tightly controlled by the lactation genome. Milk is sufficient to activate mTORC1, the crucial regulator of protein, lipid, and nucleotide synthesis orchestrating anabolism, cell growth and proliferation. To fulfill its mTORC1-activating function, milk delivers four key metabolic messengers: (1) essential branched-chain amino acids (BCAAs); (2) glutamine; (3) palmitic acid; and (4) bioactive exosomal microRNAs, which in a synergistical fashion promote mTORC1-dependent translation. In all mammals except Neolithic humans, postnatal activation of mTORC1 by milk intake is restricted to the postnatal lactation period. It is of critical concern that persistent hyperactivation of mTORC1 is associated with aging and the development of age-related disorders such as obesity, type 2 diabetes mellitus, cancer, and neurodegenerative diseases. Persistent mTORC1 activation promotes endoplasmic reticulum (ER) stress and drives an aimless quasi-program, which promotes aging and age-related diseases.

## 1. Introduction

Food is a conditioning environment that shapes the activity of the human genome [1]. Milk is an outstanding functional food developed by mammalian evolution to promote adequate growth and species-specific development of the newborn mammal. Since the Neolithic revolution, humans have consumed the milk of cows, buffalos, goats and camels during their entire lifetimes [2,3]. In the year 2011, 268 million dairy cows produced about 730 million tons of cow’s milk worldwide [4,5]. These data exemplify the enormous nutrigenomic impact of milk and dairy product consumption on human beings.

During the last decade, the understanding of milk’s biological functions has changed dramatically. Recent evidence supports the view that milk is not just a “simple food,” but that it represents a highly sophisticated endocrine signaling system that interacts with the kinase mechanistic target of rapamycin complex 1 (mTORC1). The mTORC1 pathway is the key transmitter of nutrient information to the translational machinery and is highly conserved from yeast to man [6,7,8]. mTORC1 includes the mTOR kinase, RAPTOR, mLst8, and the unique, non-conserved inhibitory subunits, DEP domain containing mTOR interacting protein (DEPTOR), and proline-rich AKT substrate of 40-kDa (PRAS40) [6,7,8]. mTORC1 mediates anabolism and the growth of the newborn mammal during the period of lactation as well as milk-driven mTORC1-dependent biological effects of the adolescent and adult human milk consumer [9]. In terms of evolutionary biology, persistent milk consumption is a novel human behavior that may exert long-term adverse effects on human health [10].

The intent of this review is to update the accumulating evidence that milk fulfills its mTORC1 activating function by providing a distinguished amino acid “hardware” and an epigenetic “software” transmitting exosomal microRNAs that modify gene expression in the milk recipient [9,11]. In a synergistic fashion, milk-derived amino acids and microRNAs modulate the magnitude of mTORC1 signaling in the milk recipient required for appropriate mTORC1-dependent translation. It is of critical concern that hyperactivated mTORC1 signaling is related to accelerated aging [12,13,14] and the pathogenesis of age-related diseases such as acne, obesity, type 2 diabetes mellitus, metabolic syndrome, cancer, and neurodegenerative diseases [15,16,17,18,19]. In fact, an epidemiological relationship between increased mortality risk and long-term milk consumption has recently been demonstrated in two large Swedish cohorts [20].

## 2. mTORC1: Key Regulator of Cell Growth and Proliferation

The nutrient-sensitive kinase mTORC1 controls cell growth and proliferation and is the central hub of metabolism that activates nucleotide, protein and lipid synthesis under conditions of nutrient and growth factor availability [21,22,23,24,25,26,27,28,29,30,31]. mTORC1 plays a pivotal role in cell cycle control and cell growth [28], protein and lipid synthesis [32,33,34,35], lipid accumulation and adipogenesis [33,34,35,36], and muscle protein synthesis [37,38,39]. Persistently overactivated mTORC1 thus increases body weight, lean and fat mass [33,34,35,36,37,38,39,40]. mTORC1 is activated by crucial nutrient-derived compounds: (1) amino acids, especially essential branched-chain amino acids (BCAAs) and glutamine; (2) growth factors, especially insulin and insulin-like growth factor-1 (IGF-1); and (3) cellular energy sources such as glucose and palmitic acid.

## 3. Amino Acids: Milk’s Hardware Activating mTORC1

Amino acid availability plays a pivotal role for the initiation of translation [41]. The branched-chain amino acids (BCAAs) leucine, isoleucine and valine are important nutrient signals for the activation of mTORC1 [23,24,25,26,27,28,29,42]. Of all amino acids, leucine plays a primary role in mTORC1 activation [43]. Leucine stimulates mTORC1 by a Rag GTP-ase dependent mechanism [44]. There is a biological reason why milk proteins such as bovine whey protein provide the highest concentrations of essential BCAAs [45]. Notably, of all animal proteins, whey proteins contain the highest amount of leucine (14%) as compared to meat (8% leucine) [45]. Furthermore, in comparison to meat, whey proteins differ remarkably in their intestinal absorption kinetics, due to their fast intestinal hydrolysis [46,47,48,49]. Thus, whey proteins operate immediately within minutes comparable to an intravenous amino acid infusion.

**Figure 1:**
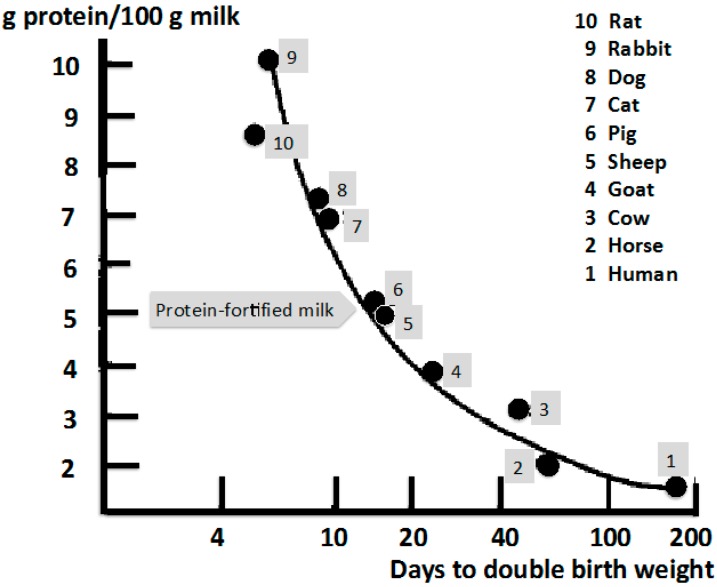
Interspecies comparison of time periods for doubling birth weight as a measure for the magnitude of mTORC1-dependent translation modified according to Bounous *et al.* [50]. Note: Human newborns (1) and other primates have the evolutionary privilege of growing on the lowest milk protein concentration (1.2 g/100 mL) thus allowing the slowest BCAA-mTORC1-driven growth, a particular advantage for complex brain development. Modern man counteracts this evolutionary privilege through uncontrolled formula feeding and persistent milk consumption. Recently, milk brands have come to market yielding milk protein concentrations artificially enriched to levels found in the milk of sheep and pig.

Remarkably, there appears to exist a “law of lactation” that predicts the growth rate of a mammalian species by determining the time required for doubling birth weight in relation to the milk protein content of the particular species [50] (Figure 1). Notably, the amount of leucine per g milk protein is a constant ratio for all mammals in the range of 10 g leucine/100 g milk protein [51] (Table 1). Thus, all mammals provide milk with a protein content of 10 percent leucine to drive mTORC1-dependent translation.

In comparison to the milk protein concentration of *Homo sapiens* (1.2 g protein/100 mL) milk of *Bos taurus* provides 3.5 g protein/100 mL. This explains why human infants double birth weight after 180 days, whereas calves double their birth weight after 40 days. This illustrates enormous differences in the magnitude and kinetics of translation and protein mass acquisition (Figure 1). In this regard, it is of critical concern that protein-enriched milk brands containing more than 5 g milk protein/100 mL have recently been introduced into the human food chain.

**Table 1:** Comparison of milk amino acid composition of selected mammalian species.

Species	Total Amino Acids * g/100 mL Whole Milk	BCAA mg/g Total Amino Acids *	Leucine mg/g Total Amino Acids *
Rat	8.69 ± 0.77	176 ± 4	92 ± 2
Cat	7.57 ± 1.27	208 ± 3	118 ± 1
Sheep	5.41 ± 0.24	196 ± 5	90 ± 4
Cow	3.36 ± 0.48	199 ± 3	99 ± 1
Horse	1.58 ± 0.35	178 ± 3	93 ± 3
Man	0.85 ± 0.09	209 ± 5	104 ± 1

Data are derived from Davis *et al.* [51]. *, Values are means ± SD of recovered amino acids excluding tryptophan. Note: For all mammals BCAA (branched-chain amino acid) account for 20% and leucine for 10% of total milk protein, respectively. Thus, the total amount of protein provided by a species’ milk determines the amount of leucine available for mTORC1-dependent translation.

Glutamine is another important activator of mTORC1 via its function as a gatekeeper for cellular leucine uptake [52,53] and via its precursor function in the glutaminolysis pathway that activates mTORC1 [54]. Leucine is an allosteric activator of glutamate dehydrogenase (GDH), the key-regulating enzyme of the glutaminolysis pathway [55,56]. It has been demonstrated in pancreatic β-cells that the interplay of glutamine and leucine maximizes the flux through GDH, which is important for mTORC1-S6K1-dependent insulin secretion [57,58]. In contrast to leucine, glutamine stimulates mTORC1 by a Rag GTPase-independent mechanism [44]. It has been demonstrated that glutamine promotes mTORC1 translocation to the lysosome in RagA and RagB knockout cells. This process requires the v-ATPase, adenosine diphosphate ribosylation factor-1 GTPase but not the RAGULATOR [44].

Bovine milk protein contains 8 g glutamine/100 g and is thus a very rich animal source of glutamine [59]. In comparison to beef protein (4.75 g glutamine/100 g), milk protein provides 70% more glutamine [59]. Leucine and glutamine represent milk’s hidden amino acid messengers. They undergo fast intestinal hydrolysis and uptake into the systemic circulation and activate mTORC1-dependent translation in a synergistic fashion. Arginine (3.11 g/100 g skim milk protein [59]) is also involved in mTORC1 activation [60]. The amino acid transporter SLC38A9 is a key component of a lysosomal membrane complex that signals arginine sufficiency to mTORC1 [60].

## 4. Milk Induces mTORC1 Activating Growth Hormones

**Figure 2:**
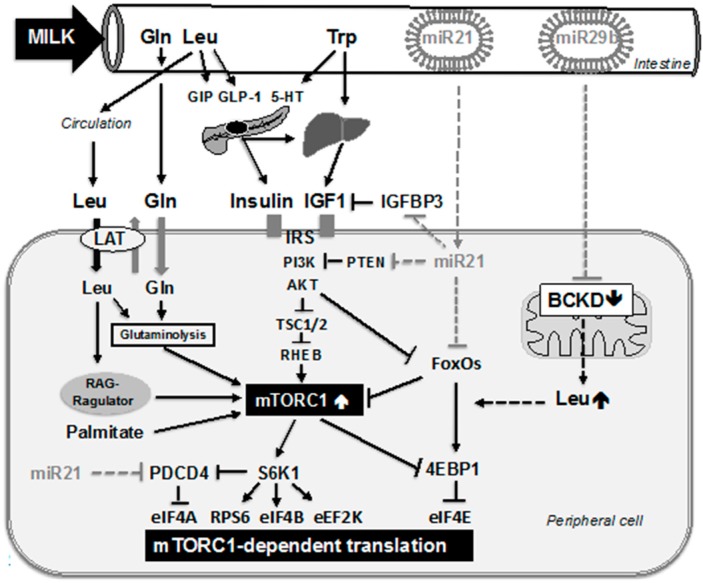
Working model of milk-induced mTORC1-dependent translation. Milk’s hardware is represented by fast-hydrolyzed amino acids leucine (Leu), glutamine (Gln) and tryptophan (Trp). Gln promotes cellular Leu uptake via the bidirectional amino acid transporter (LAT). Leu activates mTORC1 by interacting with the RAG-Ragulator complex and activates glutamate dehydrogenase, the key enzyme of glutaminolysis that activates mTORC1. Leu stimulates incretin production of glucose-dependent insulinotropic polypeptide (GIP) and glucagon-like peptide 1 (GLP-1), both stimulating insulin production. Trp via conversion to 5-hydroxytryptophan (5-HT) stimulates glucose-induced insulin secretion. Trp and insulin are required for the hepatic production of insulin-like growth factor-1 (IGF1). Insulin and IGF1 activate insulin receptor substrate (IRS), phosphoinositol-3 kinase (PI3K) and the kinase AKT, which via phosphorylation of tuberous sclerosis complex 2 (TSC2) suppresses TSC2’s inhibitory activity towards RAS-homolog enriched in brain (RHEB), the final activator of mTORC1. Milk-derived Leu, Gln and Trp and milk-derived palmitate all synergize in activating mTORC1 leading to phosphorylation of S6K1 and 4EBP1. S6K1-mediated phosphorylation of PDCD4 attenuates its inhibitory function towards eIF4A. Phosphorylation of 4EBP1 attenuates its inhibition of eIF4E. Further steps promoting translation include the S6K1-mediated phosphorylation of the translation factors RPS6, eIF4B, and eEF2K. Milk’s software program up-tuning mTORC1-dependent translation is apparently represented by milk’s exosomal microRNA-29b (miR29b) and microRNA-21 (miR21). MiR29b targets the core unit of branched-chain ketoacid dehydrogenase (BCKD), the mitochondrial key enzyme attenuating oxidative BCAA catabolism. This increases the availability of Leu for mTORC1 activation as well as synthesis of functionally important BCAA-enriched proteins. MiR21 targets IGF binding protein 3 (IGFBP3), thus increasing IGF1 bioactivity, targets phosphatase and tensin homolog (PTEN), thus activates AKT and subsequently mTORC1 and suppresses FoxO transcription factors, which are negative regulators of mTORC1. MiR21 may also affect FoxO-dependent expression of 4EBP1. MiR21 targets and inhibits the mRNA of PDCD4. PDCD4 protein is further degraded by S6K1-mediated phosphorylation. Thus, milk’s hardware and software program apparently function in a synergistic and potentiating fashion to activate mTORC1-dependent translation.

Insulin is an important growth factor that stimulates mTORC1 by activating AKT (PKB) [21,24,29]. In the pancreatic β-cell, mTORC1 plays a pivotal role in β-cell homeostasis, insulin translation, synthesis and secretion [57,61,62,63]. Milk intimately communicates with two major endocrine glands, *i.e.*, the pancreatic β-cell and the liver. Milk consumption promotes β-cell proliferation, which increases β-cell mass and thus the total insulin secretion required for insulin-driven mTORC1 activation of the peripheral somatic cells of the growing mammal [9,64,65,66,67] (Figure 2). Thus, the milk protein content is the critical determinant that promotes translation and secretion of insulin and explains why the insulinemic indices of whole cow’s milk (148 ± 14) and skim milk (140 ± 13) are nearly identical, but much higher than the glycemic indices of whole milk (42 ± 5) and skim milk (37 ± 9), respectively [68,69]. Fast hydrolysis and immediate intestinal absorption of insulinotropic amino acids of the whey protein fraction of cow’s milk raises insulin levels to much higher magnitudes than prolonged intestinal digestion of structural proteins such as beef (insulinemic index: 51) [68,69,70]. The major insulinotropic protein fraction of cow’s milk is the whey protein fraction [71]. Whey-derived leucine and other whey-derived amino acids stimulate the incretin secretion of enteroendocrine K- and L-cells, which produce glucose-dependent insulinotropic polypeptide (GIP, *syn.* gastric inhibitory polypeptide) and glucagon-like peptide 1 (GLP-1), respectively [72,73,74,75,76]. It has recently been demonstrated that the GLP-1 receptor agonist exendin-4 stimulates rodent islet cell DNA replication via activation of mTORC1 [77]. Activation of this pathway is caused by the autocrine or paracrine activation of the insulin-like growth factor 1 (IGF-1) receptor (IGF1R), as IGF1R knockdown effectively blocked exendin-4-stimulated mTORC1 activation [77].

Additionally, whey-derived amino acids directly exert insulinotropic effects on pancreatic β-cells [57,58,59,60,61,62,63]. Milk protein consumption in comparison to meat protein intake results in postprandial hyperinsulinemia [70]. Milk proteins such as α-lactalbumin provide high amounts of tryptophan, the precursor of serotonin (5-hydroxytryptophan, 5-HT). Serotonin synthesis is upregulated during pregnancy [78,79]. Commercial milk produced by pregnant cows thus contains substantial amounts of 5-HT that reach the milk consumer [80]. It has been shown that 5-HT increases β-cell proliferation and glucose-stimulated insulin secretion (GSIS) through the Gαq-coupled 5-HT2b receptor (Htr2b) and the 5-HT3 receptor (Htr3), respectively [78,79]. Thus, consumption of commercial cow’s milk provides abundant tryptophan and 5-HT that may co-stimulate GSIS of the milk recipient.

Taken together, milk provides abundant insulinotropic amino acids, especially leucine, glutamine, and tryptophan. Via stimulation of incretin secretion, direct interaction with β-cell mTORC1 signaling, and 5-HT-mediated interaction they increase insulin secretion. Insulin, a potent growth hormone, ultimately activates mTORC1 signaling of peripheral somatic cells of the milk recipient (Figure 2).

IGF-1, formerly called somatomedin C, is the most potent growth hormone that activates mTORC1 [21,22,23,27]. Milk consumption enhances hepatic synthesis and secretion of IGF-1 [71]. Growth hormone and amino acids, especially tryptophan, synergistically induce hepatic IGF-1 gene and protein expression [81,82]. Notably, the major whey protein α-lactalbumin has the highest tryptophan content among all other protein food sources [83] and after oral intake substantially increases human plasma tryptophan levels [84]. Epidemiological evidence confirms the relation between milk consumption and serum concentrations of IGF-1 [85,86]. A 20% increase in serum IGF-1 levels has been reported in prepubertal children previously not used to milk consumption after daily intake of 710 mL of ultraheat-treated (UHT) milk for 4 weeks [87]. A recent study including 193 overweight adolescents aged 12–15 years drank either 1 L/day of skimmed milk, whey, casein or water for 12 weeks [88]. All milk-based-drinks contained 35 g milk protein/L. IGF-1 significantly increased with skimmed milk and tended to increase with casein compared to the pre-test control group [88]. Casein in comparison to whey protein has been shown to differentially enhance serum IGF-1 levels [71]. Notably, per capita cheese consumption, the major dairy source of casein, increased in Germany from 5 kg in 1950 to 24.4 kg in 2013 [89]. Recent evidence indicates that glutamine controls the activity of the β-cell IGF-2/IGF1R autocrine loop by increasing the biosynthesis and secretion of IGF-2 [90]. This stimulatory effect of glutamine necessitates its metabolism but not mTORC1 activation.

Taken together, milk protein-derived amino acids such as leucine, glutamine and tryptophan are milk’s amino acid messengers that induce translation, synthesis and secretion of the mTORC1-activating growth hormones insulin, IGF-1 and IGF-2 (Table 2).

**Table 2:** Milk-derived amino acid signals that activate mTORC1-dependent translation.

Milk Amino Acid	Function	References
Leucine (Leu)	Leu stimulates intestinal production of GIP by K-cells augmenting insulin production. Insulin stimulates mTORC1 of peripheral cells of the body.	[72,75,76]
	Leu stimulates intestinal production of GLP-1 by L-cells promoting the production of insulin activating mTORC1. GLP-1 stimulates islet cell DNA replication via activation of mTORC1 involving IGF-1 signaling.	[73,75,77]
	Leu stimulates insulin production of pancreatic β-cells. Insulin stimulates mTORC1 of peripheral cells.	[57,58,62,63]
	Leu allosterically activates GDH, the rate-limiting enzyme of glutaminolysis, which activates mTORC1. GDH contributes to Leu sensing in the regulation of autophagy.	[55,56]
Glutamine (Gln)	Gln promotes cellular uptake of Leu that is the primary amino acid for mTORC1 activation. mTORC1 activation stimulates the uptake of Gln by positive regulation of glutaminase.	[52,53,91]
	Gln is the precursor of the glutaminolysis pathway that activates mTORC1 and mTORC1-dependent insulin synthesis.	[44,54,57]
	Gln controls the activity of the β-cell IGF-2/IGF1R autocrine loop by increasing biosynthesis and secretion of IGF-2.	[90]
Tryptophan (Trp)	Trp induces hepatic *IGF1* gene and IGF-1 protein expression. Insulin co-stimulates hepatic IGF-1 secretion.	[81,82]
	Trp via conversion to 5-HT enhances glucose-stimulated insulin secretion, thereby promotes mTORC1 activation.	[78,79,80]
Arginine (Arg)	The amino acid transporter SLC38A9 is a key component of a lysosomal membrane complex that signals Arg sufficiency to mTORC1.	[60]

## 5. Milk-Derived Palmitic Acid Activates mTORC1/S6K1 Signaling

It has recently been reported that the saturated C16:0 fatty acid palmitate activates mTORC1 by promoting mTORC1 activation at the lysosomal compartment [92]. Thus palmitate supports mTORC1 activation induced by BCAAs, whereas the monounsaturated fatty acid oleate (C18:1) and the ω-6 polyunsaturated fatty acid eicosapentaenoic acid (C20:5) inhibit mTORC1 activation [92]. Notably, palmitate has also been shown to upregulate mTORC1 signaling by activation of mTORC1 and its major substrate S6K1 [92,93,94,95,96]. Palmitate via activation of S6K1 enhances endoplasmic reticulum (ER) stress and induces insulin resistance [93,94,95,96], whereas inhibition of S6K1 improves insulin sensitivity [97].

Bovine milk contains about 3.5% to 5% total lipid. About 98% of the lipid is composed of triacylglycerol, transported in milk fat globules [98]. The major fatty acid of milk lipids is palmitate, representing 32.3% by weight [98,99].

Taken together, milk’s hardware is composed of essential amino acids such as leucine and glutamine and palmitate, which activate mTORC1 in the milk recipient to promote mTORC1-mediated translation. In fact, young mice fed on normal chow having additional access to cow’s milk in comparison to mice fed without milk exhibited increased mTORC1 activity and gained excessive body weight and fat mass [34].

## 6. Exosomal MicroRNAs: Milk’s Software Activating mTORC1

Valadi *et al.* were the first to demonstrate that exosome-mediated transfer of mRNAs and microRNAs is a novel mechanism of genetic exchange between cells [99]. Secreted microRNAs are now regarded as an important layer of gene regulation and intercellular communication [100,101,102,103]. MicroRNAs bind through partial sequence homology to the 3′-untranslated region (UTR) of their target mRNAs and cause either translational block or mRNA degradation [104]. Exosomal microRNAs, enclosed by membranous microvesicles, play a pivotal role for horizontal microRNA transfer [105].

Remarkably, human breast milk in comparison to all other human body fluids contains the highest amounts of total RNAs [106]. MicroRNA-containing exosomes of 30–100 nm diameter have been identified in human breast milk, cow’s milk, bovine whey and colostrum [107,108,109,110,111,112]. Bovine and human milk contain substantial amounts of exosomal microRNAs that seem to play a pivotal role in the promotion of immune regulatory functions [112,113,114,115,116]. In 2013, Melnik *et al.* hypothesized that milk functions as a “genetic transfection system” of the milk recipient by transfer of exosomal bioactive milk microRNAs to regulate the infant’s metabolic programming [9]. The authors proposed that the microRNA regulatory network represents milk’s software augmenting downstream mTORC1 signaling and cell cycle progression to optimize growth conditions of the newborn mammal during the lactation period. In fact, *in vitro* and *in vivo* evidence has recently confirmed that bovine milk-derived exosomal microRNAs reach the systemic circulation of human subjects [11] and are taken up by human mononuclear cells, macrophages as well as liver and kidney cells in culture, where they modify gene expression [11,110,111,112]. Baier *et al.* estimated that the 245 microRNAs of bovine milk modulate the transcription of more than 11,000 human genes [11]. Remarkably, bovine milk exosomes are resistant to harsh degrading conditions [111,117,118]. Raw cow’s milk contains the highest amounts of bioactive microRNAs, whereas pasteurized cooled commercial cow’s milk still contains substantial amounts of bioactive microRNAs including microRNA-29b and microRNA-21 [11,111,117,119]. As microRNA signaling is an archaic communication system of eukaryotic cells, it is not surprising that the sequences of many bovine microRNAs are identical with human microRNAs such as those of microRNA-29b and microRNA-21 (www.microrna.org).

### 6.1. Milk MicroRNA-29b: Activator of mTORC1 by Attenuating BCAA Catabolism?

The availability of BCAAs plays a fundamental role in mTORC1 activation [23,24,25,26,27,28,29,42]. The rate-controlling and irreversible step of BCAA catabolism is catalyzed by the multienzyme mitochondrial branched-chain α-ketoacid dehydrogenase (BCKD) [120] (Figure 2). About half of the BCAA catalytic activity resides in skeletal muscle, whereas a considerable portion of activity also resides in adipose tissue [120]. The BCKD complex is composed of the BCKA decarboxylase (E1), dihydrolipoyl transacylase (E2), and the dihydrolipoamide dehydrogenase (E3) [120,121]. Notably, the dihydrolipoyl transacylase (E2) forms the core of the BCKD complex [122]. In 2005, Mersey and coworkers provided evidence that human microRNA-29b controls the expression of the BCKD complex at the level of mRNA translation [123]. It is of critical concern that milk derived from cows with high lactation performance exhibits significantly higher levels of microRNA-29b in bovine mammary epithelial cells compared to cows with a lower milk yield [124]. Selection of cows with high lactation performance for commercial use may thus increase the total amount of exosomal microRNA-29 in the milk of these animals, which increases the magnitude of microRNA-29 signaling in the milk consumer. Human microRNA-29b targets the mRNA of dihydrolipoyl branched-chain acyltransferase, which forms the core of the BCKD complex and provides the binding site for all other proteins in the complex including the BCkinase [123,125]. Baier *et al.* demonstrated that bovine microRNA-29b increased in substantial amounts and in a dose-dependent manner in healthy milk consumers [11]. Furthermore, they showed that six hours after consumption of commercial pasteurized milk the intracellular microRNA-29b level doubled in peripheral blood mononuclear cells (PBMCs) of human milk consumers [11]. In fact, milk consumption evoked 30% changes in microRNA-29b target gene expression in human PBMCs such as *RUNX2* [11]. Based on these data it is reasonable to predict that milk-derived microRNA-29b also targets the mRNA of dihydrolipoyl branched-chain acyltransferase, disintegrating the BCKD metabolon of the milk consumer. This potential postnatal inhibition of BCAA catabolism during the lactation period may rescue valuable BCAAs from mitochondrial oxidation, thereby raising the BCAA serum levels required for mTORC1-dependent translation as well as synthesis of functionally and structurally BCAA-rich proteins such as hemoglobin, myoglobin, multiple enzymes, leucine zippers, surfactant protein B, and other BCAA-enriched proteins [126,127,128,129].

### 6.2. Milk MicroRNA-21 Enhances mTORC1-Dependent Translation

MicroRNA-21 is a major representative and abundant microRNA detected in raw and commercial bovine milk [111,117]. MicroRNA-21 promotes translation by activating upstream and downstream checkpoints of mTORC1 signaling. A critical well-known target of microRNA-21 is the mRNA of phosphatase and tensin homolog (PTEN) [130,131,132,133]. PTEN is a dual protein/lipid phosphatase. Its main substrate, phosphatidyl-inositol 3,4,5, triphosphate (PIP3), is the product of phosphoinositol-3 kinase (PI3K), the critical kinase activating AKT. MicroRNA-21-mediated suppression of PTEN thus promotes PI3K/AKT signaling, which downregulates nuclear forkhead box transcription factor (FoxO) activity (Figure 2). Activated AKT phosphorylates tuberosus sclerosis complex 2 (TSC2) and thereby enhances mTORC1 activity [25,29,134]. MicroRNA-21 inhibits the expression of Sprouty1 and Sprouty2 mRNA [135,136,137], a critical step that amplifies RAS-RAF-MEK-ERK signaling that suppresses TSC2 and thus increases mTORC1 activity [25]. Activated AKT phosphorylates and inactivates FoxO1 and FoxO3 by promoting their translocation from the nucleus into the cytoplasm [138,139]. Thus, microRNA-21 via attenuating PTEN decreases FoxO signaling. Furthermore, there is recent evidence that microRNA-21 directly targets mRNAs of FoxO1 and insulin-like growth factor binding protein 3 (IGFBP3) [140,141,142,143], thus decreases FoxO activity and expression. Importantly, FoxOs are negative regulators of mTORC1 signaling [144,145]. FoxO1 activates the transcription of the eukaryotic initiation factor 4 binding protein-1 (4E-BP-1), which is a major downstream substrate of mTORC1 and functions as a potent translational inhibitor and growth suppressor [146,147]. 4E-BPs inhibit translation initiation by interfering with the interaction between the cap-binding protein eIF4E and eIF4G1. Loss of this interaction diminishes the capacity of eIF4E to bind TOP and TOP-like mRNAs much more than other mRNAs, explaining why mTORC1 inhibition selectively suppresses their translation [148].

Another important target of microRNA-21 is programmed cell death 4 (PDCD4) [149,150,151] (Figure 2). PDCD4 is a suppressor of translation initiation that inhibits the RNA helicase eIF4A [152]. Both 4E-BP-1 and PDCD4 are crucial regulatory inhibitors of translation initiation and thus of protein synthesis. Activation of the mTORC1 pathway and its substrate kinase S6K1 results in subsequent phosphorylation of 4E-BP-1 and PDCD4 that promote eIF4E-eIF4G complex assembly and stimulate mRNA translation [152]. MicroRNA-21-mediated downregulation of PDCD4 amplifies translation initiation, a reasonable regulatory mode of action of milk microRNA signaling that promotes postnatal growth and anabolism (Table 3).

**Table 3:** Milk-derived exosomal microRNAs implicated in mTORC1-dependent translation.

Milk MicroRNA	Function	References
MicroRNA-29b	Targets the mRNA of dihydrolipoyl branched-chain acyltransferase, the core of the BCKD complex. This may attenuate BCAA catabolism increasing BCAA/leucine availability for mTORC1 activation.	[11,67,123]
MicroRNA-21	Targets the mRNA of IGFBP3. Resulting increase in free IGF-1 may enhance IGF-1-mediated mTORC1 activation.	[143]
MicroRNA-21	Targets the mRNA of PTEN, thus enhances the activation of AKT, which via phosphorylation of PRAS40 and TSC2 activates mTORC1 and inhibits nuclear FoxO activity. This leads to mTORC1 activation as FoxOs are negative regulators of mTORC1.	[130,131,132,133,144,145]
MicroRNA-21	Targets the mRNA of Sprouty1 and -2 thereby increasing RAS-RAF-ERK signaling. ERK-mediated phosphorylation of TSC2 activates mTORC1.	[135,136,137]
MicroRNA-21	Targets the mRNA of FoxO1, which downregulates the FoxO-promoted expression of the translational repressor 4E-BP-1.	[140,141,142,146,147]
MicroRNA-21	Targets the mRNA of PDCD4, which is a suppressor of translation initiation inhibiting the RNA helicase eIF4A.	[149,150,151,152]
MicroRNA-21	Is upregulated by TGFβ, a component of bovine milk exosomes.	[111,153]

Taken together, milk-mediated transfer of exosomal microRNA-21 apparently attenuates key inhibitory regulators of mTORC1 signaling such as PTEN, Sprouty, FoxO1, IGFBP3, and PDCD4 resulting in enhanced mTORC1-dependent translation (Table 3).

## 7. mTORC1: Key Regulator of Translation

mTORC1 plays a fundamental role in nutrient signaling and orchestrates translational regulation [41] (Figure 2). The two best-characterized downstream targets of mTORC1 are eIF4E-binding proteins (4E-BPs) and the ribosomal protein S6 kinase (S6K) [154]. 4E-BP-1 is the mTORC1 substrate that is most clearly involved in mTORC1-mediated translation [155]. 4E-BP-1 is a translational repressor that is active when hypophosphorylated and inactive when phosphorylated by mTORC1 [154,155]. mTORC1-mediated phosphorylation of S6K1 also has positive effects on translation and protein biosynthesis [154,155]. mTORC1 differentially controls the translation of specific mRNAs [148]. The subset of mRNAs that are specifically regulated by mTORC1 consists almost entirely of transcripts with established 5′ terminal oligopyrimidine (TOP) motifs or related TOP-like motifs [148]. Remarkably, loss of just the 4E-BP family of translational repressors is sufficient to render TOP and TOP-like mRNA translation resistant to the mTORC1 inhibitor Torin 1. The 4E-BPs inhibit translation initiation by interfering with the interaction between the cap-binding protein eIF4E and eIF4G1. Loss of this interaction diminishes the capacity of eIF4E to bind TOP and TOP-like mRNAs much more than other mRNAs. 4E-BP-1 represses cap-dependent translation by competing with p220 [156]. 4E-BPs and eIF4G1 are regarded as master effectors of mTORC1-controlled translation [148].

To understand the potential regulatory checkpoints of milk-driven translation, a brief presentation of the current view of translational control will be provided: The initiation stage of mRNA translation, which results in the assembly of the elongation-competent 80S ribosomes at the initiation codon, is considered to be the rate-limiting step [41,154]. Eukaryotic mRNAs possess a 5′-terminal cap structure (cap), m(7)GpppN, which facilitates ribosome binding. The cap is bound by eukaryotic translation initiation factor 4F (eIF4F), which is composed of eIF4E, eIF4G, and eIF4A. eIF4E is the cap-binding subunit, eIF4A is an RNA helicase, and eIF4G is a scaffolding protein that bridges between the mRNA and ribosome. eIF4G contains an RNA-binding domain, which was suggested to stimulate eIF4E interaction with the cap in mammals [157,158,159,160,161].

4E-BPs are the major substrate for phosphorylation by mTORC1 [155]. When nonphosphorylated (inactive mTORC1), 4E-BPs sequester the eIF4E mRNA cap-binding proteins, prevent the assembly of the eIF4F complex at the 5′-cap structure, and thereby inhibit cap-dependent mRNA translation [156]. mTORC1-mediated phosphorylation of 4E-BPs relieves their repressor function and thus promotes translation. mTORC1-mediated activation of the kinase S6K1 promotes translation through S6K1-mediated phosphorylation of eIF4B, PDCD4, eukaryotic elongation factor-2 kinase (eEF2K), and eIF3 [41,162,163] (Figure 2). Phosphorylated eIF4B promotes the helicase activity of eIF4A, which unwinds the secondary structure in the 5′-UTR of mRNAs and facilitates the scanning process of the 43S preinitiation complex [162]. When inactive, the mTORC1 substrate S6K1 is associated with the eIF3 complex. Cell stimulation promotes mTORC1 binding to the eIF3 complex and phosphorylation of S6K1 at its hydrophobic motif. Phosphorylation results in S6K1 dissociation, activation, and subsequent phosphorylation of its translational targets, including eIF4B, which is then recruited into the complex in a phosphorylation-dependent manner. Thus, the eIF3 preinitiation complex acts as a scaffold to coordinate a dynamic sequence of events in response to stimuli that promote efficient protein synthesis [163]. Tumor suppressor PDCD4 inhibits the translation initiation factor eIF4A, an RNA helicase that catalyzes the unwinding of secondary structure at the 5'-UTR of mRNAs and controls the initiation of translation (Figure 2). PDCD4 inhibits translation initiation by displacing eIF4G and RNA from eIF4A [164,165]. mTORC1 signaling via S6K1-mediated phosphorylation of PDCD4 relieves its suppression of eIF4A. Notably, S6K1-mediated phosphorylation of PDCD4 triggers its protein degradation [41].

Recently, enhancer of mRNA decapping protein 4 (Edc4) was identified as a new protein interacting with mTORC1 [166]. Decapping of mRNA, which downregulates translation, takes place in the mRNA processing body (P body) in the cytoplasm. Edc4 is an essential component for the integrity of the P body and its decapping protein complex Dcp1a and Dcp2 [167]. Whereas mTORC1-mediated phosphorylation of Edc4 inhibits the decapping machinery, its dephosphorylation activates the decapping machinery increasing mRNA degradation [166]. These observations link mTORC1 signaling to the regulation of the mRNA decapping process, which may also control microRNA-mediated translation repression [168,169].

S6K1 also affects the elongation stage of mRNA translation by phosphorylating eukaryotic elongation factor-2 kinase (eEF2K). Nonphosphorylated eEF2K negatively regulates eukaryotic elongation factor-2 (eEF2). mTORC1-driven S6K1-mediated phosphorylation of eEF2K thus activates elongation. The mTORC1-S6K1-eEF2K signaling axis has recently been shown to play a critical role in intestinal tumor initiation and growth [170].

The mTORC1/S6K1 pathway regulates glutamine metabolism through the eIF4B-dependent control of c-Myc translation [91]. Activation of the mTORC1 pathway promotes the anaplerotic entry of glutamine to the tricarboxylic acid cycle via GDH. mTORC1 activation also stimulates the uptake of glutamine by positive regulation of glutaminase through S6K1-dependent regulation of c-Myc. Notably, S6K1 enhances Myc translation efficiency by modulating the phosphorylation of eukaryotic initiation factor eIF4B [91]. In mammals, the sirtuin histone deacetylases SIRT1 and SIRT4 functionally interact with mTORC1. SIRT4 transcriptional regulation is downstream of mTORC1 signaling, and this pathway is a key regulator of glutamine metabolism [171].

Recent studies have identified additional, nuclear-specific roles for mTORC1 signaling related to transcription of the ribosomal DNA (rDNA) and ribosomal protein (RP) genes, mitotic cell cycle control, and the regulation of epigenetic processes [172]. Cyclin-dependent kinase 1 (Cdk1)-cyclin B, a key regulator complex of mitotic progression mediates protein synthesis during mitosis by controlling the activity of eEF2K [173]. Cdk1-cyclin B activity was decreased by amino acid starvation and activated by deletion of TSC2, suggesting that mTORC1 regulates Cdk1-cyclin B activity [173,174].

Taken together, mTORC1 activity modulates pivotal regulatory checkpoints controlling translation, especially the program of TOP mRNA translation. Along with mTORC1’s predominant cytoplasmic signaling function as an overseer of translation accumulating evidence underlines its direct nuclear activities as a regulator of nutrigenomics [172].

## 8. Milk’s Hardware and Software Synergize in mTORC1-Driven Translation

Amino acid and especially leucine availability is of critical importance for mammalian translation and protein biosynthesis [23,26,43,174,175,176,177]. mTORC1 signaling drives the translation of mRNAs for many anabolic enzymes and other proteins involved in diverse cellular functions [35,177]. Intracellular leucine availability and the amino acid sensor MAP4K3 are key upstream modulators of nutrient-sensitive mTORC1 signaling, whereas specific leucine metabolites or leucine oxidation rates do not play a role [176]. Notably, milk protein provides an abundant source of leucine and glutamine for intracellular leucine uptake [45,59] and via exosomal microRNA-29b transfer apparently inhibits leucine catabolism [123]. This is an important requirement for enhanced leucine-driven mTORC1 activation and subsequent stimulation of the translational machinery (Figure 2).

Milk’s leucine has thus to be regarded as a hidden mTORC1 activating messenger supported by milk-derived microRNA-29b that apparently prevents leucine catabolism [123]. Thus, milk’s “hardware” compounds leucine and glutamine combined with milk’s “software” providing microRNA-29b in a synergistic manner augment mTORC1-dependent translation.

Milk protein-derived insulinotropic amino acids establish a nutrient and signaling environment that induces mTORC1-dependent insulin production of pancreatic β-cells and hepatic IGF-1 synthesis increasing serum levels of insulin and IGF-1, respectively [21,22,23,24,27,29,57,61,62,63]. Both growth hormones via activation of the PI3K-AKT pathway stimulate mTORC1 [16,21,25] (Figure 2). AKT phosphorylates PRAS, thereby attenuating its inhibitory function against mTORC1 and phosphorylates TSC2, thereby increasing the activity of RAS-homolog enriched in brain (RHEB), that ultimately activates mTORC1 [16,21,25]. Insulinotropic amino acids, especially leucine, and IGF-1 inducing amino acids, especially tryptophan, are important nutrient signals [71] that, following swift intestinal hydrolysis, enter the systemic circulation of the milk consumer to promote mTORC1 signaling of peripheral cells of the body [9].

Exosomal milk microRNA-21 has been demonstrated in raw and commercial pasteurized cow’s milk [111,117]. As demonstrated for other exosomal milk microRNAs, milk-derived microRNA-21 may reach the systemic circulation of the milk recipient [11,111,177]. MicroRNA-21 downregulates pivotal inhibitors of upstream and downstream mTORC1 signaling: IGFBP3 [143], PTEN [130,131,132,133], Sprouty [135,136,137], FoxO1 [140,141,142], PDCD4 [149,150,151], and via FoxO suppression indirectly the expression of 4E-BP1 [146,147] (Figure 2).

Furthermore, Pieters *et al.* demonstrated that bovine milk exosomes transfer active transforming growth factor-β (TGF-β), which can induce SMAD signaling upon binding to the TGFβ receptor [111]. Notably, the TGF-β superfamily controls cell growth, differentiation, and development. SMAD proteins, the signal transducers of the TGF-β pathway, were found to regulate microRNA expression, which affects expression of numerous proteins [178]. TGFβ signaling promotes a rapid increase in the expression of mature microRNA-21 through a post-transcriptional step, promoting the processing of primary transcripts of microRNA-21 (pri-microRNA-21) into precursor microRNA-21 (pre-microRNA-21) by the DROSHA complex [153]. TGFβ-mediated microRNA expression modulates mRNA regulatory networks that determine epithelial plasticity [179]. It is thus conceivable that milk-mediated TGFβ/microRNA-21 signaling activates mTORC1, mTORC1-dependent translation and protein synthesis. However, a recent study showed that DROSHA knockdown or DICER knockout, which carry out the first and second processing steps in microRNAs biosynthesis, respectively, failed to block the translational activation of TOP mRNAs by amino acid or serum stimulation [180] pointing to a predominant role of amino acids in the promotion of translation. Currently, comparative studies of the translational efficacy of microRNA-bioactive pasteurized milk *versus* microRNA-inactivated UHT milk are missing.

Nevertheless, it has been shown that TGFβ stimulated microRNA-21 expression that induced mesangial cell hypertrophy and matrix expansion in an AKT/mTORC1-dependent manner via PTEN inhibition [132]. Neutralization of endogenous microRNA-21 abrogated TGFβ-stimulated phosphorylation of TSC2 and PRAS40, leading to inhibition of phosphorylation of S6K, mTOR and 4E-BP-1. Moreover, downregulation of microRNA-21 significantly suppressed TGFβ-induced protein synthesis and hypertrophy, which were reversed by siRNA-targeted inhibition of PTEN expression [132]. These observations clearly uncovered an essential role of TGFβ-induced expression of microRNA-21, which targets PTEN to initiate a non-canonical signaling circuit involving the AKT/mTORC1 axis for cell hypertrophy and protein synthesis [132]. Further evidence supports the pivotal role of mTORC1 for TGFβ-induced protein synthesis [181]. In renal cells, shRNA-mediated downregulation of RAPTOR inhibited TGFβ-stimulated mTOR kinase activity, resulting in inhibition of phosphorylation of S6K and 4E-BP-1 [181]. TGFβ, a component of bovine milk exosomes [111], thus plays a crucial role in regulating the RAPTOR-RICTOR axis of protein synthesis [181]. Importantly, TGFβ reduced DEPTOR levels in a time-dependent manner with concomitant increase in both mTORC1 and mTORC2 activities. Expression of DEPTOR abrogated activity of mTORC1 and mTORC2, resulting in inhibition of collagen I (α2) mRNA and protein expression via transcriptional mechanism [182]. TGFβ-stimulated SMAD3 contributes to DEPTOR suppression and mTORC1 activation [183].

Taken together, substantial evidence points to a crucial role of milk-derived exosomes in microRNA-21 and TGFβ/microRNA-21-mediated upregulation of mTORC1-dependent translation.

## 9. Milk-Mediated Over-Activation of mTORC1 and Age-Related Diseases

**Figure 3:**
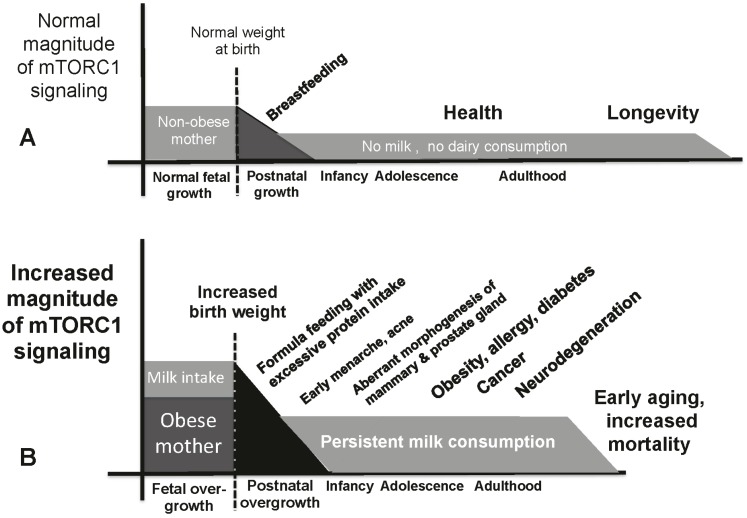
Synoptic model illustrates the influence of milk signaling on the magnitude of mTORC1 activation. (**A**) Physiological milk-driven mTORC1 signaling. Pregnancy without maternal overweight and with milk consumption allows regular fetal growth and normal birth weight. Breastfeeding guarantees lactation genome-controlled adjustment of the appropriate postnatal mTORC1-signaling axis for physiological metabolic programming; (**B**) Maternal obesity and milk consumption during pregnancy promote fetal overgrowth and macrosomia. Artificial formula feeding with excessive protein intake further amplifies aberrant mTORC1-dependent metabolic programming. Persistent milk consumption ultimately consolidates aberrant hyperactivation of mTORC1, a quasi program that accelerates aging and early onset of age-related diseases.

Increased mTORC1 signaling has been related to the pathogenesis of Western diseases such as obesity, type 2 diabetes, cancer and neurodegenerative disorders [13,14,15,16,17,18,19]. Persistent lifetime exposure to cow’s milk signaling from the beginning of intrauterine life to adulthood is a novel human behavior introduced by the Neolithic revolution and boosted by the widespread availability of refrigerator technology since the early 1950s, which allows daily access to milk’s essential BCAAs and exosomal microRNAs provided by commercial pasteurized cow’s milk. Accumulating evidence underlines that milk consumption during pregnancy increases fetal growth and birth weight [184,185,186], which is the result of abundant uptake of BCAAs and microRNA-21 promoting placental and fetal overgrowth (Figure 3). There is an emerging role of mTORC1 signaling in placental nutrient sensing [187,188,189,190]. Leucine availability plays a critical role for placental mTORC1 activity and mTORC1-mediated trophoblast BCAA transfer to the fetus promoting translation and protein synthesis of fetal cells. It is conceivable that bovine milk exosomal microRNA-21 reaches the placenta and contributes to placental and fetal overgrowth [190]. In fact, aberrant upregulation of microRNA-21 has been observed in placental tissue of infants with macrosomia [191]. It has been shown in rats that maternal overweight induced by a high fat diet activates placental mTORC1 and eIF2α signaling and increases fetal growth [192]. Thus, maternal overweight combined with milk consumption during pregnancy and subsequent formula feeding with excessive protein intake are most critical constellations programming adipogenesis and lifetime obesity [186] (Figure 3).

Consumption of commercial pasteurized milk of young mice increased mTORC1 signaling, weight gain and fat mass accretion [34]. Evidence has been provided that mTORC1 signaling is increased in obesity, suppresses lipolysis, stimulates lipogenesis and promotes fat storage [32,33,36,193]. Indeed, milk consumption in children, adolescents and adults has been demonstrated to increase BMI and fat mass [194,195,196,197,198,199].

Remarkably, recent epidemiological data exhibit an increased association of obesity and allergy [200,201,202,203]. Obesity and allergy/asthma development have both been related to accelerated growth trajectories early in infancy [204,205,206,207,208]. A key feature of allergy and asthma is the deficiency of FoxP3^+^ regulatory T-cells (Tregs) [209]. Low mTORC1 activity is required for the differentiation FoxP3^+^ Tregs [210,211,212]. It has been demonstrated that increased AKT-mTORC1 signaling negatively regulates *de novo* differentiation of CD4^+^FoxP3^+^ Tregs in the thymus and downregulates FoxP3 expression in peripheral tissues [213,214,215,216]. Increased mTORC1 signaling during early infancy, a period frequently associated with increased milk consumption, thus explains the molecular link between hyperactivated mTORC1 and the development and comorbidity of obesity and allergy [217]. Noteworthy, elevated serum levels of microRNA-21 have been detected in asthma patients [218].

Milk consumption not only enhances adipocyte lipid synthesis but also sebocyte lipid production linking milk consumption to the development of the mTORC1-driven skin disease *acne vulgaris* [219,220,221,222,223,224,225]. This common and often diet-induced inflammatory skin disease of Western civilization does not exist in populations living under Paleolithic conditions without exposure to milk, dairy and hyperglycemic carbohydrates [226].

Remarkably, milk consumption in children accelerates the onset of puberty and has been related to early onset of menarche [227], which is an epidemiological risk factor for the development of obesity, insulin resistance and type 2 diabetes mellitus [228,229,230]. The largest worldwide study investigating the type of dairy product intake and incident type 2 diabetes mellitus is the EPIC-InterAct study [231], a nested case cohort within eight European countries (*n* = 340,234). Although, the pooled hazard ratios (HRs) demonstrated only a slight but significant increase of diabetes risk in relation to increased milk intake, HRs of individual country cohorts showed substantial variations exhibiting higher diabetes risks in the French, Italian, UK, German and Swedish cohorts. mTORC1 signaling is required for β-cell mass regulation and insulin production [63,232], the major secretory protein synthesized by β-cells. Overstimulated mTORC1-driven insulin synthesis by persistent milk consumption may enhance β-cell ER stress promoting early β-cell apoptosis and accelerating the onset of type 2 diabetes mellitus [233,234,235,236,237,238,239,240]. Notably, impaired IRS signaling in transgenic mice overexpressing S6K in β-cells resulted in impaired β-cell survival [241]. In accordance with S6K, the mTORC1 substrate 4E-BP-1, a key inhibitor of translation initiation, is also involved in the regulation of β-cell survival. Intriguingly, deletion of 4E-BP-1 in a mouse model of diabetes accelerated β-cell loss [242]. Krokowski *et al.* recently demonstrated that a self-defeating anabolic program leads to β-cell apoptosis in ER stress-induced diabetes via regulation of amino acid flux [243]. Thus, persistent milk-driven β-cell mTORC1 signaling with overactivated insulin translation appears to promote early β-cell apoptosis as recently demonstrated in a rodent model with overstimulated mTORC1 signaling [244].

mTORC1, the master regulator of protein synthesis, couples nutrient sensing to cell growth and cancer [245]. It has been demonstrated in prostate cancer cells that the translational landscape of mTOR signaling steers cancer initiation and metastasis [148,245,246,247,248]. Emerging evidence demonstrates a key role for the PI3K-AKT-mTORC1 signaling axis in the development and maintenance of castration-resistant prostate cancer [249]. Notably, mTOR regulates epithelial-mesenchymal transition at least in part by downregulation of RhoA and Rac1 signaling pathways [249]. Prostate tumor overexpressed-1 (PTOV1), a modulator of the Mediator transcriptional regulatory complex, is expressed at high levels in prostate cancer and other neoplasias in association with more aggressive disease [250]. PTOV1 was associated with ribosomes and its overexpression promoted global protein synthesis in prostate cancer cells and COS-7 fibroblasts in a mTORC1-dependent manner [250]. There is compelling epidemiological evidence that whole milk intake is associated with prostate cancer-specific mortality among U.S. male physicians [251]. The European Prospective Investigation into Cancer and Nutrition (EPIC), which studied 142,251 men, estimated that a 35 g/day increase in the consumption of dairy protein was associated with a 32% increase in the risk of prostate cancer [252]. Importantly, daily milk consumption during adolescence (*vs.* less than daily), but not in midlife or currently, was associated with a 3.2-fold risk of advanced prostate cancer [253], pointing to a critical signaling magnitude of mTORC1-dependent prostate branching morphogenesis during adolescence [254], the prostate’s period of sexual maturation [246]. There is accumulating evidence that microRNA-21 contributes to prostate cancer pathogenesis interfering with multiple pathways including mTORC1-dependent translation and cell proliferation [255,256,257,258]. In fact, addition of commercial cows' milk to LNCaP prostate cancer cells stimulated growth of prostate cancer cells and increased the growth rate of the tumor cells over 30% [259]. In contrast, a recent feeding study, which unfortunately used powdered milk (apparently without any bioactive microRNAs) exhibited no effect on hyperplasia or neoplasia in two mouse models of fully penetrant genetically-induced prostate tumorigenesis (probasin-Prl mice, Pb-Prl and KIMAP mice) [260]. Noteworthy, this study did not reflect the physiological amino acid-microRNA interplay of whole milk signaling.

During a 7 to 13 years prospective follow-up study, 248 of 25,892 Norwegian women developed breast cancer. Notably, women consuming more than 750 mL of whole milk on a daily basis had a relative breast cancer risk of 2.91 compared with those who consumed 150 mL or less [261]. There is further experimental evidence that milk consumption promotes breast cancer. Feeding of commercial cow’s milk compared to a milk-free diet doubled mammary tumor mass and numbers in 7,12-dimethylbenz(a)anthracene-induced mammary tumors in rats [262]. Furthermore, milk feeding inhibited the regression of 7,12-dimethylbenz(a)anthracene-induced mammary tumors in ovariectomized rats [263]. As in the prostate’s gland, puberty initiates branching morphogenesis of the mammary gland, which requires growth hormone, estrogen, as well as IGF-1, to create a ductal tree that fills the fat pad [264]. In analogy with the pathogenesis of prostate cancer, increased milk-derived IGF-1 signaling may disturb regular mammary gland morphogenesis enhancing the risk of breast cancer later in life. Increased serum levels of IGF-1 have been associated with a high risk of both prostate and breast cancer [265,266,267,268]. Circulating serum IGF-1 levels, which are upregulated during puberty and by milk consumption [71,85,86,87,88], have been related to mammographic density, a well-known risk factor of breast cancer [269,270]. IGF-1 induces anterior gradient 2 (AGR2) in the breast cancer MCF7 cell line, through an estrogen response element and a leucine zipper transcription factor-binding site on the AGR2 promoter playing a key role in IGF-1-induced breast cancer cell proliferation and migration [271].

Worster *et al.* found that the abundance of the cyclin-dependent kinase inhibitors p21Cip1 and p57Kip2 increased in response to IGF-1 or insulin but decreased in response to EGF [272]. Depletion of p57Kip2 but not p21Cip1 rendered IGF-1 or insulin sufficient to induce cellular proliferation in the absence of EGF. Remarkably, microRNA-21, a major microRNA of commercial milk [111,117], has recently been shown to target p57Kip2 in prostate cancer cells [256]. Increased levels of microRNA-21 have been found in serum and mammary tumor tissue of breast cancer patients [273,274,275,276]. Knockdown of p57Kip2 enhanced the proliferative phenotype induced by tumor-associated PI3K mutant variants and released mammary epithelial acini from growth arrest during morphogenesis in three-dimensional culture [272]. Furthermore, TGFβ, a component of bovine milk exosomes [111], has been demonstrated to promote mammary tumorigenesis permitting late-stage breast cancer cells to acquire an invasive and metastatic phenotype [277]. Higher mTOR expression was noted in breast cancer tissue, higher grade tumors, in ductal tumors, and was associated with worse overall survival [278]. Furthermore, the expression of the mTORC1 component RAPTOR was associated with a higher tumor grade. A highly significant positive correlation between mTORC1 and hTERT, the catalytic subunit of telomerase was recently observed [278]. Thus, mTORC1 is an important upregulator of telomerase in breast cancer, which contributes to the carcinogenic effects of increased IGF-1/mTORC1 signaling. In contrast to milk, metformin treatment of patients with diabetes mellitus type 2 significantly reduced breast cancer risk [279,280,281]. Accumulating evidence supports the view that metformin is a multiple-layer mTORC1 inhibitor as reviewed by Melnik and Schmitz [282].

Recent evidence points to a crucial role of environmental and dietary factors in the pathogenesis of the neurodegenerative disorders such as Parkinson’s and Alzheimer’s disease [283]. Epidemiological evidence linked milk consumption to increased risk of Parkinson’s disease [284,285,286]. A Chinese meta-analysis confirmed a dose-response relationship with a 17% increased risk of Parkinson’s disease for every daily 200 g increment in milk intake [284]. Importantly, Alzheimer’s and Parkinson’s diseases are tauopathies that exhibit increased mTORC1-mediated phosphorylation of tau proteins resulting in tau protein dyshomeostasis [18,285,286,287,288]. Pathological hyperphosphorylated tau aggregates to form neurofibrillary tangles. Tang *et al.* showed that mTORC1 mediates the synthesis and aggregation of tau [19]. Sun *et al.* demonstrated that mTORC1 was activated in the hippocampus region of patients with Alzheimer’s disease [287]. Note that the level of mTORC1 activation correlates with cognitive deficiencies of these patients [287]. In contrast, the mTORC1 inhibitor rapamycin decreased tau synthesis [19,288] and increased cognitive functions in animal models of Alzheimer’s disease [289,290]. As with rapamycin, the mTORC1 inhibitor metformin decreases tau phosphorylation via mTOR/protein phosphatase 2A (PP2A) signaling [291].

Taken together, persistent milk-driven overactivation of TORC1 signaling represents the mechanistic link to mTORC1-mediated diseases of civilization.

## 10. Conclusions

Milk is a masterpiece of mammalian evolution, guaranteeing the well-regulated growth with appropriate growth velocity of the particular species essential for ordered postnatal programming. This mammary gland secretory product is tightly controlled by the lactation genome of the corresponding species and maintains the required magnitude of mTORC1-dependent translation. Milk is thus the optimized nutrient and signaling system for nutrigenomic regulation during the lactation period. Milk plays an exceptional role in the beginning of mammalian life and performs its biological function by delivering its amino acid hardware and exosomal microRNA software. These messengers of milk have only one primary mission: to activate and maintain mTORC1-dependent translation and other mTORC1-mediated anabolic effects during the period of postnatal growth and postnatal metabolic programming [9,292].

Mammary gland-derived exosomes transmit a sophisticated array of microRNAs that function as a “Trojan horse”, like a retrovirus infection, to “transfect“ the newborn infant with maternal microRNAs that modify infant’s gene expression at the level of posttranscriptional regulation [9,293,294]. In this context, milk is best viewed as each mammalian mother’s nutrigenomic doping system, accelerating postnatal anabolism, cell growth, and cell proliferation of the offspring. Unfortunately, pediatricians of the 1930s were unable to recognize milk’s mTORC1-dependent functions and introduced artificial uncontrolled protein-rich formula for infant feeding [295]. This obviously did not match the physiological lactation genome-controlled mTORC1-signaling axis of human breast milk and thus initiated the epidemic of obesity [292]. American pediatric specialist McKim Marriott declared “There is nothing mysterious and sacred about breast milk, it is just food. It is perfectly possible to prepare an artificial formula which meets all the nutritional requirements” [296]. This severe misinterpretation of milk’s nature dramatically overstimulated postnatal mTORC1 signaling resulting in significant increases in BMI and fat mass of formula-fed infants.

Further technical progress of the 1950s resulted in widespread distribution of refrigerators that allowed daily access to pasteurized cow’s milk and a steady increasing variety of other BCAA-rich dairy products. This technological “progress” dramatically modified the human food exposome and introduced unnoticed bioactive bovine microRNA transfer into the human food chain. From that time on, modern humans were persistently exposed to the gene-regulatory network of bovine milk, the secretory product of a fourtimes faster growing mammalian species.

Daily consumption of cooled pasteurized milk thus allows excessive intake of milk’s amino acid hardware and milk’s gene-regulatory software, which in a synergistic fashion upregulate mTORC1 signaling enhancing mTORC1-dependent anabolism and mTORC1-dependent mRNA translation. It is becoming apparent that this unnoticed modification of epigenetics by milk consumption has had an enormous impact on modern human nutrigenomics 10,000 years since the Neolithic revolution. While the early Neolithic period was characterized by the consumption of fermented milk and milk products with inactivated bovine microRNAs, the introduction of modern cooling facilities and large scale pasteurization of milk exposed industrialized societies to the synergistic interplay of milk’s amino acid hardware and its bioactive exosomal software.

In 2014, a leading US company launched marketing of a protein-enriched milk (5.4 g milk protein/100 mL) compared to regular cow’s milk (3.5 g/100 mL). This latest achievement of Western civilization was made possible by technical skills in milk ultrafiltration and recombination of milk ingredients. Luckily, humans had not yet developed artificial milk providing 10 g milk protein/100 mL as present in the milk of rats, which double their birth weight already by five days of age [50].

Permanent overactivation of mTORC1 signaling is the key mechanism driving mTORC1-mediated age-related diseases of civilization [16,17,18,19,67,89,287,293]. In this regard, the observed milk-related increase in mortality recently reported in two large Swedish cohorts [20] is well explained by milk’s biological function as a driver of mTORC1 accelerating ageing and age-related diseases that increase overall mortality. Already in 1934, McCay and Crowell recognized by methods of comparative biology that slowing growth favors longevity in various animal species [297]. It is thus no surprise that mTORC1-driven comorbidities such as type 2 diabetes and ischemic heart disease are associated with milk consumption [298], whereas populations with a high (>30%) prevalence of lactose malabsorption, whose milk intake is low, had a lower risk of ischemic heart disease [298]. In another recently published Swedish cohort study, people with low milk consumption due to lactose intolerance exhibited a decreased risk of lung, breast, and ovarian cancers [299].

Recent evidence has been provided that the widely prescribed anti-diabetic drug metformin functions as inhibitor of mTORC1 signaling [282]. Intriguingly, metformin not only improves type 2 diabetes but decreases the risk of cancer [279,280,300], and apparently prolongs lifespan in humans [301]. Metformin thus counteracts milk-driven activation of mTORC1 signaling and mTORC1-driven translation [282].

We are still at the very beginning of understanding milk’s epigenetic regulatory network. MicroRNA-21 and -29b, two striking milk-transmitted microRNAs, are obviously involved in the upregulation of mTORC1-dependent translation. It has been estimated that daily access to bioactive bovine microRNAs affects more than 11,000 human genes [11], which may have major nutrigenomic impacts on the process of ageing and the age-related pathologies that are not yet recognized.

The study of the Karolinska Institute of Michaëlsson *et al.* [20] elicited a controversial debate concerning the role of milk consumption in human health [302]. Unfortunately, the investigators tried to explain the adverse effects of milk consumption as due to increased galactose-mediated oxidative stress and increased proinflammatory signaling supported by milk-related increases of serum interleukin 6 (IL-6) levels [20]. Note that Sweden belongs to the top 10 countries consuming primarily pasteurized cow’s milk containing bioactive microRNAs [11,111,119]. Recent evidence demonstrates that overexpression of microRNA-21, one of milk’s major microRNA constituents, reduces replicative lifespan [303], while microRNA-21 knockdown extended the cell’s replicative lifespan. In fact, it has been shown that reduction of microRNA-21 in U87 and LN229 glioblastoma cells repressed STAT3 expression and STAT3 phosphorylation [304]. Thus, increased microRNA-21 signaling induced by consumption of pasteurized milk may explain a nutrigenomic constellation that increased STAT3-mediated IL-6 expression. Notably, increased microRNA-21 serum levels have been found in various conditions of chronic inflammation and obesity [305,306,307,308,309], whereas long-term inhibition of microRNA-21 reduced obesity in db/db mice [310].

Half a century ago, the antagonistic pleiotropy theory of aging postulated that natural selection favors genes that are beneficial early in life, even if they cause aging later in life [311,312]. mTOR is absolutely beneficial and necessary early in life but operates later at the cost of aging [306]. Early in life, mTORC1 drives developmental programs, which persist later in life as aimless, indeed misdirected quasi-programs of aging and age-related diseases [307]. According to Blagosklonny, aging is not and cannot be programmed. Instead, aging is a continuation of developmental growth, driven by genetic pathways such as mTORC1 signaling [312]. Milk is mammals’ superior, most sophisticated and extremely robust mTORC1-driver activating developmental growth for survival of mammalian species. However, beyond the physiological period of lactation persistent milk consumption operates as a superfluous quasi-program promoting age-related mTORC1-driven diseases of civilization (Figure 3). In this regard, the recently launched production and commercialization of ultrafiltered pasteurized milk brands containing artificially enriched milk protein content (5.4 g protein/100 mL) compared to regular commercial milk (3.3 g protein/100 mL) is the most recent and most critical nutritional aberration since the Neolithic revolution. According to George Martin “The brightest flame casts the darkest shadow” [313]. Translated in terms of molecular medicine this means: the higher the magnitude of mTORC1 signaling, the earlier we encounter aging and age-related pathologies [312]. Persistent milk signaling leads to alterations in cell homeostasis, ER stress, cellular malfunctions, organ damage and thus early onset of age-related diseases. In order to prevent aging and age-related diseases Kapahi *et al.* emphasized “With TOR less is more” [314]. The recent fascinating progress in mTORC1 biology allows a deeper understanding of the adverse effects of persistent milk consumption on human health. Future nutritional studies will need to differentiate total amino acid content, and their bioavailability, total microRNA content, transfer and bioactivity of milk and milk products. Additional critical factors are the impact of milk processing, fermentation and fractionation [11,46,47,48,49,108,109,110,111,112,113,114,115,116,117,118,119,315].

Persistent abuse of a developmental nutrient and programming system of another mammal such as *Bos taurus*, a species whose initial growth rate is four times that of humans, is thus a major pathogenic factor promoting the epidemic diseases of civilization [316]. Wiley was right when she pointed out that persistent cow’s milk consumption is a novel human behavior potentially exerting long-term adverse effects on human health [10]. Taken together: “No milk today, that’s what this message means, the end of obese and Western disease!”.
